# ﻿Revalidation of *Passalites* Gloger, 1841 for the Amazon brown brocket deer *P.nemorivagus* (Cuvier, 1817) (Mammalia, Artiodactyla, Cervidae)

**DOI:** 10.3897/zookeys.1167.100577

**Published:** 2023-06-20

**Authors:** Jorge Alfonso Morales-Donoso, Gabrielle Queiroz Vacari, Agda Maria Bernegossi, Eluzai Dinai Pinto Sandoval, Pedro Henrique Faria Peres, David Javier Galindo, Benoit de Thoisy, Miluse Vozdova, Svatava Kubickova, José Mauricio Barbanti Duarte

**Affiliations:** 1 Núcleo de Pesquisa e Conservação de Cervídeos (NUPECCE), Departamento de Zootecnia, Faculdade de Ciências Agrárias e Veterinárias, Universidade Estadual Paulista (UNESP), Jaboticabal-SP, Brazil Universidade Estadual Paulista (UNESP) Jaboticabal Brazil; 2 Laboratorio de Reproducción Animal, Departamento de Producción Animal, Facultad de Medicina Veterinaria, Universidad Nacional Mayor de San Marco, San Borja, Lima, Peru Universidad Nacional Mayor de San Marco Lima Peru; 3 Kwata NGO, Cayenne, French Guiana Kwata NGO Cayenne French Guiana; 4 Central European Institute of Technology-Veterinary Research Institute, 621 00, Brno, Czech Republic Central European Institute of Technology-Veterinary Research Institute Brno Czech Republic

**Keywords:** brocket deer, Cervidae, *Mazamanemorivaga*, new genus, taxonomy

## Abstract

*Mazamanemorivaga* (Cuvier, 1817) is a gray brocket deer that inhabits the Amazon region. An assessment of previous studies revealed inconsistencies in its current taxonomic classification, suggesting the need for an update in its genus classification. A taxonomic repositioning of this species is proposed through the collection of a specimen from its type locality (French Guiana) with subsequent morphological (coloring pattern, body measurements, and craniometry), cytogenetics (G Band, C Band, conventional Giemsa, Ag-NOR staining, and BAC probe mapping), and molecular phylogenetic analysis (mitochondrial genes Cyt B of 920 bp, COI I of 658 bp, D-loop 610 bp), and comparisons with other specimens of the same taxon, as well as other Neotropical deer species. The morphological and cytogenetic differences between this and other Neotropical Cervidae confirm the taxon as a unique and valid species. The phylogenetic analysis evidenced the basal position of the *M.nemorivaga* specimens within the Blastocerina clade. This shows early diversification and wide divergence from the other species, suggesting that the taxon should be transferred to a different genus. A taxonomic update of the genus name is proposed through the validation of *Passalites* Gloger, 1841, with *Passalitesnemorivagus* (Cuvier, 1817) as the type species. Future research should focus on evaluating the potential existence of other species within the genus *Passalites*, as suggested in the literature.

## ﻿Introduction

Brocket deer, genera *Mazama* and *Subulo*, are widely distributed and occur in almost every tropical and subtropical forest region between south-central Mexico (State of Veracruz) and northern Argentina (Czernay 1987; Duarte and Gonzalez 2010). Its main characteristics are a small to medium body size with unbranched and straight, skewer-shaped, antlers (Rossi 2000). Taxonomic problems have previously been pointed out in the genus *Mazama*, regarding species validity and diversity (Duarte et al. 2008; Gutiérrez et al. 2017). Brocket deer have little morphological differentiation (Groves and Grubb 1987) that are not correlated to their wide karyotypic diversity (Duarte and Merino 1997; Duarte 1998; Duarte et al. 2008). As a polyphyletic group, the genus *Mazama* is considered one of the most impressive and surprising cases of morphological convergence among mammals (Duarte et al. 2008). Thus, many doubts remain about the taxonomic status of the species composing this group (Gilbert et al. 2006; Duarte et al. 2008; Gutiérrez et al. 2017).

The polyphyletic status of the genus *Mazama* has been suggested since the early 2000s by studies applying molecular phylogenetic analyses using sequences of the mitochondrial cytochrome *b* gene and the nuclear genes MGF and IL16 (Gilbert et al. 2006). The species traditionally included in genus *Mazama* are distributed in two subtribes, the Odocoileina with *Mazamaamericana* (Erxleben, 1777), *Mazamajucunda* Thomas, 1913, *Mazamanana* (Hensel, 1872), *Mazamatemama* (Kerr, 1792), *Mazamarufa* (Illiger, 1815), and *Mazamarufina* (Pucheran, 1851), and the Blastocerina, with the species *Subulogouazoubira* (Fischer, 1814), *Mazamachunyi* (Hershkovitz, 1959) and *Mazamanemorivaga* (Cuvier, 1817) (Gilbert et al. 2006; Duarte et al. 2008; Hassanin et al. 2012; Heckeberg et al. 2016; Gutiérrez et al. 2017). Once *Mazamarufa* was selected as the type species of the genus (Merriam 1895), the two Blastocerina lineages should be allocated to other genera. This was already done for *Mazamagouazoubira*, which was transferred to the genus *Subulo* Smith, 1827 (Bernegossi et al. 2022a).

By contrast, *M.nemorivaga* is a lineage that still deserves a taxonomic assessment. The species is the smallest gray brocket deer occurring in the Amazon region (Rossi and Duarte 2008; Rossi et al. 2010). Based on field observations and museum specimens, its geographic distribution includes Guyana, French Guiana, Suriname, Venezuela, Colombia, Panama, Ecuador, Peru, Brazil, and probably Bolivia (Rossi et al. 2010). In addition to the original Amazonian distribution, its occurrence in the Atlantic Forest was suggested by Rossi (2000) and recently confirmed by the detection of populations on the eastern coast of Brazil (Oliveira et al. 2020).

The species was originally described as *Cervusnemorivagus* by Cuvier (1817) from specimens collected by Poiteau and Martin in Cayenne, French Guiana and stored at the National Museum of Natural History in Paris (MNHN) (Pucheran 1852). Despite historical indications that the holotype would be found at the MNHN (Brooke 1878) and that a taxidermized specimen collected by Poiteau still exists in the museum, it is not possible to affirm whether this specimen was evaluated by Cuvier, since the author did not explicitly indicate the specimens examined (pers. comm. Cécile Callou, 24 June 2015).

The taxonomic literature on New World deer has historically alternated between validating and synonymizing several species of gray/brown brocket deer of smaller size than red brockets. *Mazamanemorivaga* has always been considered as a valid taxon in revisions considering more than one species of this morphotype (up to seven species of brocket deer have been recognized) (Brooke 1878; Lydekker 1898; Allen 1915). After Cabrera’s revision in 1960, *S.gouazoubira* was commonly recognized as the only gray brocket species, while *M.nemorivaga* and other taxa were treated either as synonyms or as subspecies (Cabrera 1960; Czernay 1987; Duarte and Merino 1997). The taxonomic review of the Brazilian brocket deer species conducted by Rossi (2000) once again recognized the validity of *M.nemorivaga* based on morphological patterns such as coat color and cranial dimensions that were described as diagnostic for the species when compared with other brockets. Furthermore, studies on reproductive isolation between *Mazamanemorivaga* and *Subulogouazoubira* suggested some degree of pre-mating isolation (Carranza et al. 2017) and strong post-mating isolation due to hybrid infertility (Galindo et al. 2021b).

To date, 34 synonyms for *M.nemorivaga* have been listed. Of these, 13 present insufficient descriptions, and their association with either *M.nemorivaga* or *S.gouazoubira* is uncertain (Rossi et al. 2010). Several authors considered seven of these synonyms as potential *S.gouazoubira* subspecies. Now they represent potential *M.nemorivaga* subspecies (Cabrera 1960; Hershkovitz 1951; Czernay 1987; Pinder and Leeuwenberg 1997; Medellin et al. 1998). This is the case for *M.americanacitus* Osgood, 1912, *M.gouazoubiramedemi* Barriga-Bonilla, 1966, *Mazamamurelia* Allen, 1915, *Mazamapermira* Kellogg, 1946, *Mazamarondoni* Miranda-Ribeiro, 1914, *Mazamacitasanctaemartae* Allen, 1915, and *Cervustschudii* Wagner, 1855. In addition to subspecies recognition, some of these named forms may represent valid species. Due to the low levels of morphological differentiation among brocket deer, karyotypic analyses have proven an especially important tool for defining the *Mazama* species (Duarte and Jorge 1996, 2003; Abril and Duarte 2008; Cifuentes-Rincón et al. 2020, Bernegossi et al. 2022a). *Mazamanemorivaga* has shown intraspecific karyotypic variation with diploid numbers between 66 and 70, a fundamental number between 70 and 72, and a simple or multiple sexual system depending on the absence or presence of an X autosomal fusion, respectively (Duarte and Merino 1997; Duarte 1998; Rossi et al. 2010; Fiorillo et al. 2013). Individuals from the Brazilian states of Mato Grosso, Pará, Maranhão, Amapá, Rondônia, and Acre were analyzed in these studies. The extensive degree of polymorphism in this species suggests a cryptic species complex reproductively isolated by chromosomal differences (Fiorillo et al. 2013; Galindo et al. 2021a).

As morphological (Rossi 2000), cytogenetic (Fiorillo et al. 2013; Bernegossi et al. 2022a), and molecular studies (Duarte et al. 2008; Hassanin et al. 2012; Heckeberg et al. 2016; Gutiérrez et al. 2017) suggest that the current classification of *M.nemorivaga* has inconsistencies, the integrative characterization of a recently collected topotype is warranted. As such, this study characterized *M.nemorivaga* through morphological analysis (body biometry, color patterns, craniometry, post-skull characterization), cytogenetics (conventional karyotype, chromosomal biometry, C-banding, G-banding, Ag-NOR-staining, BAC, and bovine chromosomal probes), and molecular analyses (three mitochondrial regions). The sensu stricto species definition based on these morphological and genetic patterns recovered suggest the existence of other species that should be separated from this taxon. We also formally transfer this species to the genus *Passalites* Gloger, 1841, as has already been suggested in previous studies. This taxonomic action is an important milestone for the phylogenetic relationships among cervids to be properly considered in systematics.

## ﻿Materials and methods

### ﻿Collection of topotype and morphology

We collected an adult male topotype in the city of Régina, 70 km south-east of Cayenne in French Guiana, allowed by the collection permission document n°2014328-0018 issued by the Prfet de la Rgion Guyane, Direction de l’Environnement, de l’Aménagement et du Logement and by approval n°005433/19 from the UNESP/Jaboticabal ethics committee. In accordance with the Access and Benefit Sharing requirement, a tissue sample is also deposited in French Guiana (collection JAGUARS, Kwata NGO) under the reference number M5865_JAG. The specimen was photographed, and 14 external body measurements were taken using a caliper (head width, distance between the eyes, width from the jaw to its base), a measuring tape (head length, neck, thorax, and abdomen circumference; body, ear, tail, metatarsal and metacarpal length, and height), and a scale (body mass). The complete skeleton and the taxidermized skin were deposited at the Museum of the Deer Research and Conservation Center (NUPECCE - Núcleo de Pesquisa e Conservação de Cervídeos), in Jaboticabal, São Paulo, Brazil, under the catalog number NPC080. Aspects such as general coat coloration, body chromogenetic fields, band pigment pattern on hairs, and the presence of anteverted hairs and tufts were also examined. Additionally, head chromogenetic fields were examined according to the pattern described by Hershkovitz (1982). The skull was photographed at different angles, and 38 measurements were taken according to the measurement standard proposed by Rees (1969) and von den Driesch (1976) with a digital caliper (0.1 mm).

A matrix was constructed with the cranial measurements of the French Guiana topotype and of other Neotropical adult deer deposited in the NUPECCE Museum, corresponding to the species *P.nemorivagus*, *S.gouazoubira*, *M.americana*, *M.rufa*, *M.nana*, and *M.jucunda* (Table 1). All individuals used in the matrix were adult males of each of the previously mentioned species in order to avoid the effects of sexual dimorphism as indicated by González et al. (2018). In order to condense the morphological variables into five factors, we performed a factorial analysis using the VARIMAX method followed by a principal component analysis (PCA) to analyze similarity patterns between individuals and evaluate a possible differentiation of the species. All analyses were performed using the “Paleontological Statistics” PAST software (Hammer et al. 2001).

**Table 1.** Specimens used in morphological, cytogenetic, and molecular analyses (BD = body measurement).

Voucher ID	Species	Origin	Karyotype	Skull	BD	Mitochondrial DNA			Reference
						Cytb	COI	D-loop	
T359	*P.nemorivagus*	Régina, French Guiana (topotype of *nemorivaga*)	2n = 69 and FN = 72, XY	X	X	MT008225	OQ918560	OQ923298	This study
T309	*P.nemorivagus*	Amapá, Brazil	2n = 68 and FN = 72, XY	–	–	MT008223	OQ918557	OQ923295	This study
T346	*P.nemorivagus*	Amapá, Brazil	2n = 68 and FN = 72, XY	–	–	MZ350867	OQ918559	OQ923297	This study
T321	*P.nemorivagus*	Macapá, Brazil	2n = 69 and FN = 72, XY	–	–	MT008224	OQ918558	OQ923296	This study
JN632660	*P.nemorivagus*	French Guiana	–	–	–	JN632660	JN632660	JN632660	Hassanin et al. 2012
T377	*S.gouazoubira*	Puerto Galileo, Paraguay (neotype of *guazoubira*)	–	X	–	MZ350858	MZ350858	MZ350858	Bernegossi et al. 2022a
T082	*S.gouazoubira*	Camobi, Rio Grande do Sul, Brazil	–	X	–	MZ350862	MZ350862	MZ350862	Bernegossi et al. 2022a
T386	*S.gouazoubira*	Matão, São Paulo, Brazil	–	X	–	MZ350865	MZ350865	MZ350865	Bernegossi et al. 2022a
KJ772514	*S.gouazoubira*	Pantanal, Brazil	–	–	–	KJ772514	KJ772514	KJ772514	Caparroz et al. 2015
T389	*S.gouazoubira*	Puerto Arecutacuá, Paraguay	–	X	–	MZ350866	MZ350866	MZ350866	Bernegossi et al. 2022a
NC020682	*B.dichotomus*	–	–	–	–	NC020642	NC020642	NC020642	Hassanin et al. 2012
JN632603	*B.dichotomus*	Bolívia	–	–	–	JN632603	JN632603	JN632603	Hassanin et al. 2012
DQ789193	*O.bezoarticus*	Bolívia	–	–	–	DQ789193	DQ789193	DQ789193	Duarte et al. 2008
JN632657	*M.americana*	Peru	–	–	–	JN632657	JN632657	JN632657	Hassanin et al. 2012
JN632656	*M.americana*	French Guiana	–	–	–	JN632656	JN632656	JN632656	Hassanin et al. 2012
T358	*M.americana*	French Guiana (neotype of *americana*)	–	X	–	MZ350857	MZ350857	MZ350857	Bernegossi et al. 2022a
T253	*M.americana*	Juína, Brazil	–	X	–	MZ350856	MZ350856	MZ350856	Bernegossi et al. 2022a
T107	*M.nana*	Paraguay	–	–	–	MZ350863	MZ350863	MZ350863	Bernegossi et al. 2022a
T215	*M.jucunda*	P. E. Intervales-SP, Brazil	–	–	–	MZ350859	MZ350859	MZ350859	Bernegossi et al. 2022a
T362	*M.temama*	Campeche, México	–	–	–	MZ362858	MZ362858	MZ362858	Bernegossi et al. 2022a
T366	*M.temama*	Veracruz, México (neotype of *temama*)	–	–	–	MZ350864	MZ350864	MZ350864	Bernegossi et al. 2022a
MF784604	*A.alces*	–	–	–	–	MF784604	MF784604	MF784604	Świsłocka et al. 2020
MN813763	*C.pygargus*	–	–	–	–	MN813763	MN813763	MN813763	Ao et al. 2020
MT753444	*R.tarandus*	–	–	–	–	MT753444	MT753444	MT753444	Artyushin et al. 2020
CATFAP4	*P.nemorivagus*	Captivity, Brazil	–	X	–	–	–	–	This study
T371	*P.nemorivagus*	Pará, Brazil	–	X	–	–	–	–	This study
T264	*P.nemorivagus*	Pará, Brazil	–	X	–	–	–	–	This study
T265	*P.nemorivagus*	Maranhão, Brazil	–	X	–	–	–	–	This study
T347B	*S.gouazoubira*	São Paulo, Brazil	–	X	–	–	–	–	This study
T403	*S.gouazoubira*	Santa Catarina, Brazil	–	X	–	–	–	–	This study
T323	*S.gouazoubira*	São Paulo, Brazil	–	X	–	–	–	–	This study
T409	*S.gouazoubira*	Brazil	–	X	–	–	–	–	This study
T260	*M.americana*	Santarém, Brazil	–	X	–	–	–	–	This study
T259	*M.americana*	Santarém, Brazil	–	X	–	–	–	–	This study
T247	*M.americana*	Juína, Brazil	–	X	–	–	–	–	This study
T251	*M.americana*	Juína, Brazil	–	X	–	–	–	–	This study
T269	*M.americana*	Rondônia, Brazil	–	X	–	–	–	–	This study
T206	*M.americana*	Rondônia, Brazil	–	X	–	–	–	–	This study
T205	*M.rufa*	Paraná, Brazil	–	X	–	–	–	–	This study
T268	*M.rufa*	Paraná, Brazil	–	X	–	–	–	–	This study
T304	*M.nana*	Rio Grande do Sul, Brazil	–	X	–	–	–	–	This study
NPC114	*M.nana*	Brazil	–	X	–	–	–	–	This study
T412	*M.jucunda*	Paraná, Brazil	–	X	–	–	–	–	This study
T340	*M.jucunda*	Paraná, Brazil	–	X	–	–	–	–	This study

### ﻿Cytogenetic characterization

Trichotomy and antisepsis were performed on the inner region of the left thigh, followed by the excision of a 2 × 2 cm skin fragment, which was then preserved in liquid nitrogen as described in Duarte et al. (2021). Subsequently, fragments were thawed and cultivated in order to obtain fibroblastic lineages for chromosomal preparations (Verma and Babu 1995). Chromosome preparations were subjected to G-banding (Seabright 1971), C-banding (Sumner 1972), and Ag-NOR staining (Howell and Black 1980). Chromosomes were classified as metacentric, submetacentric, or acrocentric based on the arm length ratio. Through relative length (RL), autosomal chromosomes were classified as group A (large two-armed chromosomes, with CR > 6%), C (small two-armed chromosomes, with CR < 6%), D (large acrocentric chromosomes, with CR > 5%), E (small acrocentric chromosomes, with CR < 5%), and B (B chromosomes, with CR < 1.5%) (Cifuentes-Rincón et al. 2020). In addition to the topotype, we cytogenetically evaluated three other *P.nemorivagus* belonging to the NUPECCE sample bank collected from near the region of the type locality in order to verify if there were any significant chromosomal differences among specimens (Table 1).

### ﻿Fluorescent in situ hybridization

We mapped bovine-derived artificial bacterial chromosome (BAC) probes into the *P.nemorivagus* topotype. Probes were selected considering the mapped *S.gouazoubira* karyotype (Bernegossi et al. 2022b). BACs were obtained from the CHORI-240 bovine library based on NCBI ARS-UCD1.2 assembly data from BACPAC Genomics, Emeryville, CA, USA (Suppl. material 1). For DNA extraction, a protocol adapted from the method included in the Wizard® Plus SV Minipreps DNA Purification Systems was used and labeling was performed with Green-dUTP (Abbott, IL, USA), 16-dUTP biotin or digoxigenin-11-dUTP (Roche, Mannheim, Germany) using a CGH Genomic Labeling BioPrime® Array (Invitrogen, Carlsbad, CA, USA). Fluorescent *in situ* hybridization was performed according to Vozdova et al. (2019). Slides were analyzed on a Zeiss Axio Imager Z2 fluorescence microscope (Carl Zeiss Microimaging GmbH, Jena, Germany). Metafer Slide Scanning System software (MetaSystems, Altlussheim, Germany) was used to locate and capture metaphase images. All images were edited using Adobe Photoshop CS2.

### ﻿Molecular characterization

The mitochondrial DNA sequences of 24 deer species were used in this study (Table 1). These were generated in the NUPECCE and downloaded from GenBank (http:\\www.ncbi.nlm.nih.gov\genbank).

### ﻿DNA extraction

Genomic DNA extraction from tissue samples (liver and muscle) was performed by proteinase K digestion and a phenol/chloroform extraction through a modified protocol based on the methodology described by Sambrook et al. (1989). Samples were quantified by spectrophotometry (Eppendorf BioPhotometer®) and then diluted in water or TE at use concentration (100 ng/µl) after a concentration reading in a spectrophotometer.

### ﻿DNA amplification and sequencing

We amplified the following partial gene sequences: 920 bp of cytochrome *b* (Cyt B) (Hassanin et al. 1998; Duarte et al. 2008), 658 bp of the cytochrome *c* oxidase subunit I (COI I) (Folmer et al. 1994; Hassanin and Ropiquet 2004), and 610 bp of the D-loop region (Vilá et al. 1999) (Suppl. material 2).

Samples were submitted to PCR in a conventional thermocycler “Biometer T1 Thermocycler” for DNA amplification. DNA fragments were amplified using 300 μM dNTPs, 1.5 mM MgCl, 1x Buffer (200 mM Tris-HCl pH 8.4; 500 mM KCl), 1U Taq Polymerase, 15–20 pmol forward primer, 15–20 pmol of reverse primer, 50–100 ng DNA, in a final volume of 30 µL. The amplification program was an initial 5-min cycle at 94° for DNA denaturation; 35 cycles at 94° for 1 min with Cyt-B and COI I hybridization temperature at 54°, and 52° for the D-loop primer, and 45–75 sec at 72° for 1 min, with a final extension of 72° for 30 min.

After PCR, the amplified product was analyzed by 2% agarose gel electrophoresis and purified by an ethanol protocol without washing (Dorado-Pérez 2012). The PCR products were sequenced using the ABI Prism Big DyeTerminator kit (Applied Biosystems ®), and the final product was analyzed with an ABI Prism 377 automatic sequencer (Applied Biosystems ®).

### ﻿Model selection and phylogenetic analysis

The sense and antisense sequences of the mitochondrial regions were visually inspected on electropherograms in order to eliminate false polymorphisms and possible ambiguities and a combined into a consensus sequence using the BioEdit Sequence Alignment Editor software (Hall 1999).

The evolutionary model with the best fit to our sequence data was estimated through Partitionfinder2 using the CIPRES Science Gateway platform (Miller et al. 2010). Phylogenetic analysis was performed using Bayesian inference through the MrBayes v. 3.2.1 program in XSEDE, available as a webservice on the CIPRES Science Gateway platform (Miller et al 2010). The analysis included two runs and four Markov Chain Monte Carlo (MCMC) chains with 10,000,000 generations each and a 25% burn-in. The tree obtained from the analysis was edited using the program FigTree v. 1.4.0 (Rambaut 2012).

## ﻿Results

### ﻿Description of the male *Passalitesnemorivagus* (Cuvier, 1817) topotype

**Figure 1.**
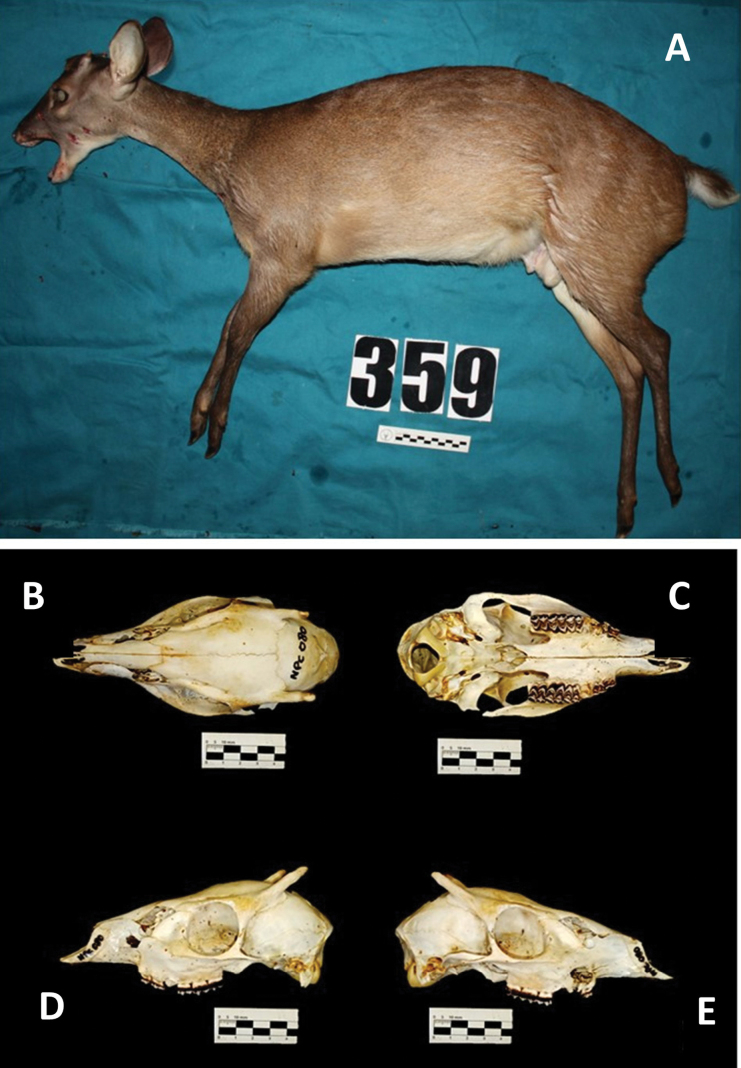
Adult male *Passalitesnemorivagus* (Cuvier, 1817) topotype from Régina, French Guiana (T359) **A** lateral view of the topotype body **B–E** skull in different views **B** dorsal **C** ventral **D** left side **E** right side. Other photographs of the topotype are illustrated in Suppl. material 5.

General orange-brown coloration, dorsally dark brown uniform. Flanks light brown to brown. Tail, dorsally brown, ventrally white. Head, neck, and chest region from dark to light brown. Absence of anteverted hair strip on nape. White nasal spot, light brown rostral lateral band, deep brown rostral band, brown inferior orbital band over a yellowish spot, orange superior orbital band, whitish pre-orbital gland opening. Mental spot present, yellowish mandibular spot, and white buccal and throat region. White basal auricular spot, white inner auricular surface, and orange-brown outer auricular surface. Presence of a tuft of frontal hair. Absence of tuft of hairs on tarsal region. Whitish inguinal and abdominal region. Brown lateral region of the limbs. Internal distal region of the limbs brown on the hind limbs and orange-brown on the forelegs. Whitish inner proximal region of the limbs. Skull with absent sagittal crest, small tympanic bulla, extended vomerinus septum, two lacrimal foramina at the edge of the orbit, shallow lacrimal fossa, and rectangular pre-orbital region (Fig. 1).

### ﻿Morphometry

External body measurements obtained from the topotype are listed in Table 2.

**Table 2.** Body measurements of the *Passalitesnemorivagus* topotype, collected in French Guiana, measurements in centimeters (cm) and mass in kilograms (kg).

Character	Measurement
Head length	20.5
Head width	7.55
Ear length	8.6
Between eyes	4.5
Mandible width (basis)	5.3
Metacarpus length	12.5
Height	48.0
Body length	70.0
Tail length	9.0
Metatarsus length	21.0
Neck Circumference	21.0
Thorax circumference	54.0
Abdomen circumference	63.0
Body mass	14.5

**Figure 2.**
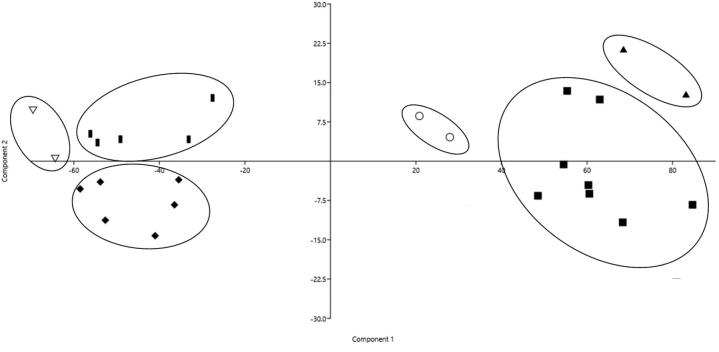
Principal component analysis (PCA) of adult male *P.nemorivagus* (Cuvier, 1817) (filled diamonds), *S.gouazoubira* (Fischer, 1814) (filled bars), *M.nana* (Hensel, 1872) (inverted triangles), *M.rufa* (Illiger, 1815) (filled triangles), *M.jucunda* Thomas, 1913 (circles), and *M.americana* (Erxleben, 1777) sensu lato (filled squares) cranial measurements.

Cranial dimension factorial analysis simplified 38 measurements into four factors. Factors 1 and 2 are associated with skull length. Measurements include total length (TL), condylobasal length (CBL), short skull length (SSL), basefacial axis (BFA), vicerocranial length (VCL), nasal lambda (NL), lambda-prostion (LP), oral palatal length (OPL), and short facial length (SFL). Factor 3 is associated with mean frontal length (MFL), akrocranium (ACR), and tooth running distance (TRD). Factor 4 is related to skull width measurements such as the nasal bone’s most distal region (NBDR), zygomatic width (ZW), greater mastoid width (GMW), and premolar line (PML). Principal component analysis of these cranial measurements showed that PC1 and PC2 (principal components 1 and 2, respectively) represented 95.64% of the data variance, with length measurements influencing PC1 (X axis) and shape measures influencing PC2 (Y axis). The analysis efficiently discriminated the specimens primarily by size, where medium-sized individuals corresponded to *M.americanasensu lato*, *M.rufa*, and *M.jucunda*, and were recovered separately from small individuals corresponding to *P.nemorivagus*, *S.gouazoubira*, and *M.nana* (Fig. 2). Also, the analysis evidenced the similarity of these measurements between individuals of the same species, forming clusters of inter-specific differentiation. Specifically, the *P.nemorivagus* specimens, including the topotype of the present study, were recovered in a cluster separated from *Subulogouazoubira* and other species of the *Mazama* genus. All morphometric data are available in the supplementary material (Suppl. material 3).

### ﻿Cytogenetic analyses

**Figure 3.**
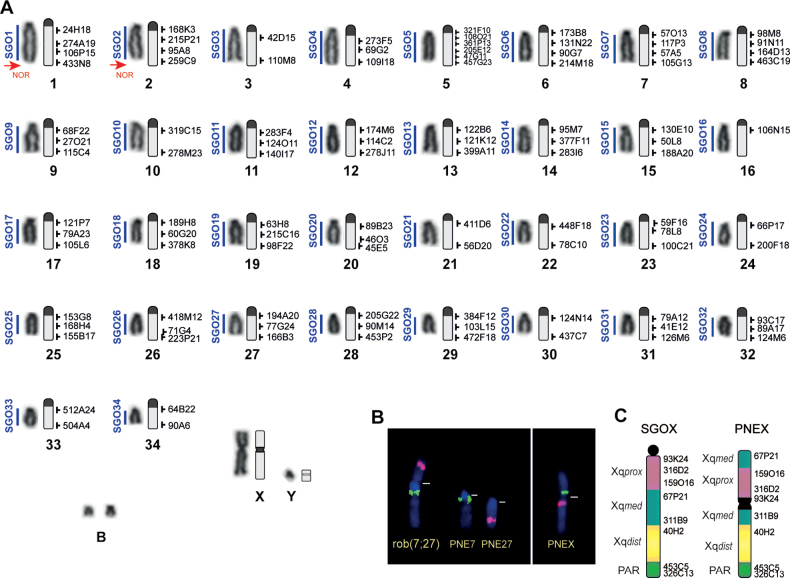
**A** from left to right – homologous *Subulogouazoubira* chromosomes (Fischer, 1814) (SGO); chromosomes of the male *Passalitesnemorivagus* topotype (Cuvier, 1817) (PNE) under conventional Giemsa staining; C-banding idiogram, demonstrating heterochromatin blocks; and labeling selected BAC probes from the CHORI-240 library. Arrows indicate Ag-NOR staining **B** FISH showing the chromosomes involved in the Robertsonian translocation (rob)7;27; and probes 311B9 (pink) and 316D2 (green) on the PNEX**C** Schematic comparison of the BAC probe markings on the X chromosome between SGO and PNE. Xqprox = proximal X region; Xqmed = medial X region; Xqdist = distal X region; PAR = Psedoautosomal region.

The collected specimen showed a karyotypic constitution with a diploid number (2n) of 69 chromosomes and a fundamental arm number (FN) of 72 (Fig. 3). Autosomal chromosome pairs were all acrocentric, with the exception of one submetacentric chromosome, which was the product of a centric fusion in the heterozygous condition. Chromosomal measurements by relative length (RL) classified all chromosomes into Group E (small acrocentric chromosomes). Both X and Y sex chromosomes were submetacentric. The number of B chromosomes ranged from 0 to 3; these were the smallest chromosomes in the lot.

C-banding showed that all autosomal chromosomes had pericentromeric blocks of constitutive heterochromatin. The X chromosome showed a heterochromatic block in the centromeric region, and the Y chromosome was euchromatic. B chromosomes, on the other hand, were heterochromatic. Ag-NOR staining revealed nucleolar organizer regions in the telomere region of the first two chromosome pairs.

G-banding patterns and BAC probe staining were used for comparison with *S.gouazoubira* (SGO), which retained the ancestral karyotype of the species. We observed that centric fusion in heterozygosis involved pairs 7 and 27 of the topotype. The *P.nemorivagus* X chromosome (PNEX) differs from SGOX by a pericentric inversion and a change in the centromeric position, resulting in the submetacentric morphology of the PNEX. All other chromosomes matched in terms of morphology, banding pattern, and order of BAC probes when compared with the ancestral karyotype (SGO). The comparative analysis with other *P.nemorivagus* specimens showed that all individuals have fusions in chromosome pairs 7 and 27. The topotype (T359), and the individual T321 carry the fusion in heterozygosis, while the individuals T309 and T346 carry the fusion in homozygosis. All other chromosomes from the autosomal set have one homologous chromosome. The sexual system was simple (XY) for all individuals analyzed.

### ﻿Phylogenetic characterization

**Figure 4.**
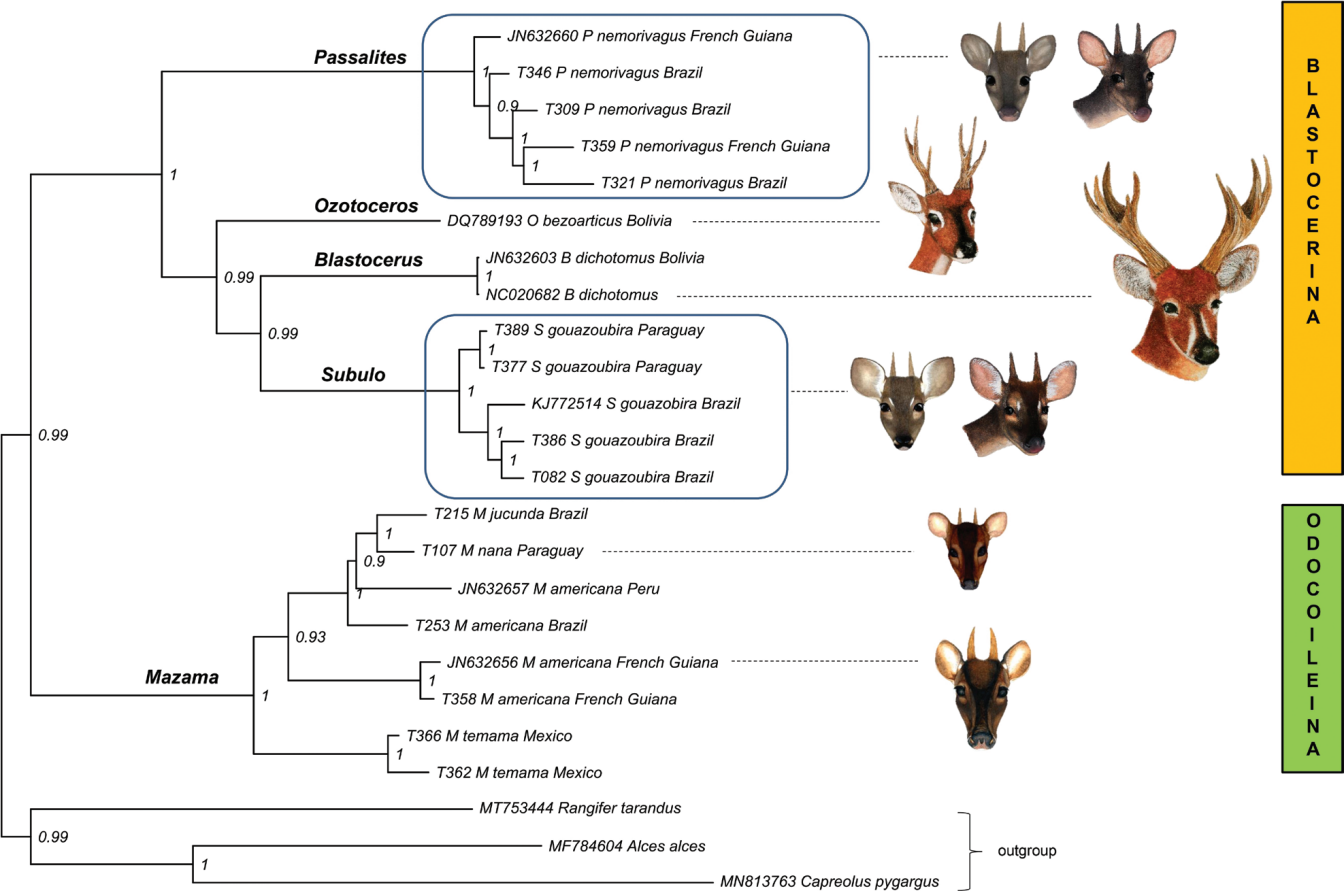
Phylogenetic tree based on Bayesian Inference from concatenated mitochondrial DNA regions of cytochrome *b* (Cyt-B, 920 pb), Cytochrome *c* oxidase subunit I (COI, 658 pb), and the control region (D-loop, 610 pb).

The analyses conducted with the three concatenated mitochondrial gene regions (Cyt-B; COI I; D-loop) recovered *P.nemorivagus* in a very well-supported clade and sister group to the other Blastocerina (Fig. 4). Moreover, the genetic pairwise Kimura 2-parameter distance of *Passalitesnemorivagus* compared to other Neotropical deer is available in Suppl. material 4.

## ﻿Discussion

The designation of a new genus for *Mazamanemorivaga* (Cuvier, 1817) has been suggested since the first phylogenetic review included this species (Duarte et al. 2008). Subsequent authors reaffirmed the error in using the genus name *Mazama* for this taxon (Hassanin et al. 2012; Heckeberg et al. 2016; Gutiérrez et al. 2017). *Passalitesnemorivagus* is allocated within the subtribe Blastocerina, while the *Mazama* type species, *M.rufa* (Illiger, 1815), is recovered within the Odocoileina subtribe (Heckeberg et al. 2016). This result has been confirmed in the phylogenetic analysis of the present study, which included a topotype of the species. Thus, we propose to allocate *nemorivagus* in a genus distinct from *Mazama*.

### ﻿Taxonomic updating of generic and specific names

The first description of a New World brocket deer within Linnaeus’s binomial system allocated the red brocket deer to the genus *Moschus* L. (*Moschusamericanus* Erxleben, 1777), a group that included the other small ungulates already known to science at the time, such as the chevrotains (Erxleben 1777). Shortly afterwards, Kerr (1792) described several species, placing them in the genus *Cervus* L., where European deer were traditionally classified. Despite the proposition of new names, many of the new species’ classifications and descriptions maintained the use of *Cervus* L. for decades (e.g., Illiger 1815; Lesson 1842; Pucheran 1851). The first genus name proposed exclusively for the brocket deer was *Mazama* Rafinesque, 1817. The origin of the name came from the Nahuatl word for “deer” in Mexico, as reported by Hernandez (1651) as “mazame,” “maçame,” or “teuthlamaçame”. In fact, Kerr (1792) suggested *Cervustemama* for the first brocket deer described in Mexico in allusion to its popular names. The description of the genus *Mazama* was made in three subsequent editions by Rafinesque in the year 1817. In the first (September), the author originally considered the species *Mazamapita* [= *M.rufa* (Illiger, 1815)] and *Mazamabira* [= *S.gouazoubira* (Fischer, 1814)]. The second (October) lists *Mazamapudu* [= *Pudupuda* (Molina, 1872)], *Mazamaorina*, and *Mazamacaprina* [= *Antilocapraamericana* (Ord, 1815)]. Finally, in the last one (November), the formal description of the genus considers only the Mexican species *Mazamatema* [=*M.temama* (Kerr, 1792)], and the mountain goats *Mazamadorsata* and *Mazamasericea* [= *Antilocapraamericana* (Ord 1815)], the latter being the version that had been adopted for the longest (Palmer 1904). The *M.rufa* type species was fixed only later by Merriam (1895), who rescued the genus as the oldest available name for the brocket deer given the first publication made by Rafinesque (1817) in September, solving the confusion regarding its application to branched-antlered deer (Smith 1827) and mountain goats (Saussure 1860; Palmer 1904).

The synonymy of *Mazama* Rafinesque, 1817 is well known and consistent across several studies (Merriam 1895; Allen 1915; Cabrera 1960; Rossi 2000; Varela et al. 2010; Jasper et al. 2022). The second proposed genus name for the brocket deer is Subulo Smith, 1827, which was proposed as a subgenus and would include all brocket deer species known at the time, Cervus (Subulo) rufus Illiger, 1815, Cervus (Subulo) simplicicornis Illiger, 1815, and Cervus (Subulo) nemorivagus Cuvier, 1817. At the time, the type species had not been designated. After several studies indicating the polyphyletic condition of the genus *Mazama* and the existence of three independent lineages (Gilbert et al 2006; Duarte et al 2008; Gutiérrez et al 2017), Bernegossi et al. (2022a) suggested the name *Subulo* to designate the *S.gouazoubira* lineage (Fischer, 1814). Following Smith’s review (1827), Gloger’s work (1841) proposes the genus *Passalites* with *P.nemorivagus* as the only species (Cuvier, 1817). Therefore, the type species of *Passalites* Gloger, 1841 is *Cervusnemorivagus* Cuvier, 1817 by monotypy. The genus name is one of the exceptions listed in Article 30.1.4.4 of the International Code of Zoological Nomenclature in which Greek names ending with the suffix “-*ites*” are to be treated as masculine unless the original author has explicitly stated otherwise (ICZN, 4^th^ edition). Thus, we understand that the combination *Passalitesnemorivagus* (Cuvier, 1817) should be used in the present case.

***Passalites* Gloger, 1841**

**Etymology of genus name.** From the Greek *πασσαλος*, meaning skewer, which characterizes their simple, unbranched, spiked antlers.

**Type species of the genus.***Cervusnemorivagus* Cuvier, 1817, by monotypy (Gloger, 1841).

**Species included in the genus.** Only the type species.

**Generic synonymy.**

*Cervus*: Cuvier, 1817: 485. Part, not *Cervus* Linnaeus, 1758.

*Mazama*: Lydekker, 1898: 303. Part, not *Mazama* Rafinesque, 1817.

*Subulo* Smith, 1827: 319. Part, restricted to *Subulogouazoubira* (Fischer, 1814) by Bernegossi et al. 2022.

*Coassus* Gray, 1843: 174. No type species selected.

*Dorycerus* Fitzinger, 1873: 360. No type species selected.

*Cariacus*: Brooke, 1878: 918. Part, not *Cariacus* Lesson, 1842.

*Hippocamelus*: Elliot, 1907: 50 Part, not *Hippocamelus* Leuckart, 1816.

**Revised diagnosis.** Phylogenetically defined as a clade of brocket deer in the Blastocerina sub-tribe, Odocoileini tribe of the Cervidae family (Fig. 3), distinguishable from other spike-antlered deer through a set of morphological features summarized by Rossi et al. (2010) from the comparative analyses in Duarte (1996), Medellín et al. (1998), and Rossi (2000). According to Rossi et al. (2010), *Passalitesnemorivagus* (Cuvier, 1817), the only representative of the genus *Passalites* Gloger, 1841, has a grayish brown coat without any shade of red that allows its diagnosis in relation to the sympatric red brocket deer *Mazamaamericana* (Erxleben, 1777) and most other species now recognized in the *Mazama* Rafinesque, 1817 genus (*M.rufa*; *M.jucunda*; *M.nana*; *M.rufina*; *M.temama*). It differs from *M.chunyi* by its considerably larger size and from *M.pandora* by the narrower postorbital constriction, slender pedicels, and parallel antlers in contrast to broader postorbital constriction, massive pedicels, and divergent antlers (Medellín et al. 1998). Finally, the differentiation of *Passalites* in relation to the genus *Subulo* Smith, 1827 – also a member of the Blastocerina sub-tribe – and its only species, *S.gouazoubira* (Fischer, 1814) is also possible given the presence of smaller and pointed ears, larger eyes, larger orbital cavities, and narrower auditory bulla in *P.nemorivagus* compared with larger, rounded ears, smaller eyes, smaller orbital cavities, and wider auditory bulla in *S.gouazoubira* (Duarte 1996; Rossi 2000). González et al. (2018) showed craniometric differences between *P.nemorivagus* and *S.gouazoubira*, the latter being on average 5% larger in all measurements, with the exception of premolar-prosthion, basifacial axis, and least breadth between orbits, which were larger in *P.nemorivagus* males and females. Furthermore, both species have a karyotype with 2n = 70, but the FN = 70 in *S.gouazoubira* (Bernegossi et al. 2022a) and FN = 72 in *P.nemorivagus*. The FN divergence is related to a morphological difference of the X sexual chromosome, which is acrocentric in *S.gouazoubira* (Bernegossi et al. 2022a) and submetacentric in *P.nemorivagus*, while all autosomes are acrocentric in the two species.

### ﻿*Passalitesnemorivagus* (Cuvier, 1817)

#### Topotype assignment

The animal described here can be considered a topotype for *P.nemorivagus*, representing a comparative baseline for future taxonomic revisions. There are indications that the taxidermized specimen from the National Museum of Natural History in Paris was part of the original set used for the formal description of the species and should thus be considered a syntype. The animal presented here is the first specimen analyzed with a reliable origin, having been collected near the type locality in a region of continuous forest. Its morphological description corresponds with the original description by Cuvier (1817) in relation to the small body size, the grayish brown coloration, the detailing of white regions below the tail and throat, and the whitish and cinnamon-tinged outline of the eye (Cuvier 1817). Furthermore, despite some doubt regarding the presence of more than one species of red brocket, there has never been a suggestion on the occurrence of two species of small brown brocket in the Guiana region (Tate 1939; Hollowell and Reynolds 2005).

#### Morphology, cytogenetic, and DNA findings

The coloration and external body measurements of the *P.nemorivagus* topotype corroborate the described diagnostic characters reported in previous studies, showing that the species corresponds to a small gray deer with large eyes and pointed ears (Rossi 2000; Rossi et al. 2010). A uniform gray coloration was observed in all specimens in the study, the only difference being associated with the sharpness of the yellowish and orange hairs in the topotype’s orbital region differentiated from the generally brown blurred pattern reported in other descriptions, confirming the individual variability of this character (Rossi 2000).

Just as coat color is the main differentiating feature between *P.nemorivagus* and *Mazama*, external body measurements and craniometry have been reported to be discriminatory with *S.gouazoubira* (González et al. 2018). Morphometric analyses corroborate that the small size in *P.nemorivagus* is associated with smaller dimensions in most measurements (González et al. 2018), except for orbital height and width (OH and OW), which were larger than in *S.gouazoubira* (Rossi 2000). Thus, in addition to proving the efficiency in discriminating species through cranial characters, the factor analysis and subsequent principal component analysis (PCA) support the hypothesis that *P.nemorivagus* is a morphometrically distinct species from *S.gouazoubira* (González et al. 2018).

Regarding the cytogenetic characterization of the species, previous descriptions demonstrated the existence of a variation of 2n = 66 to 70 and FN = 70 to 72 which are associated with centric fusions and/or X-autosomal fusions (Resende 2012; Fiorillo et al. 2013). As such, the topotype showed a heterozygous fusion between the PNE7 and PNE27 autosomes. We observed that this fusion was also present in all studied individuals, in either homozygous or heterozygous form. However, we did not observe X-autosomal fusions as described in Fiorillo et al. (2013) for *P.nemorivagus* individuals from the Amazon´s northeast region. It is possible that this fusion with the sex chromosome is exclusive to populations from the south of the Amazon River (Fiorillo et al. 2013). The X-autosomal fusion can lead to a decrease in reproductive efficiency when populations that do not carry it come into contact with carriers and reproduce, potentially resulting in postzygotic reproductive isolation (Galindo et al. 2021a). Moreover, the BAC map produced for the topotype may serve as a basis for comparison of other *P.nemorivagus* chromosomal variants, both for identification of the chromosome involving X-autosomal fusions and for postzygotic reproductive barrier studies using *sperm*-FISH with individuals born from crosses between different chromosomal lineages (Galindo et al. 2021a, b).

The cytogenetic comparison between *P.nemorivagus* and *S.gouazoubira* demonstrated that their autosomal sets present the same morphological and banding pattern; however, the X chromosome of *P.nemorivagus* has a submetacentric morphology, while the X chromosome is acrocentric in *S.gouazoubira* (Bernegossi et al. 2022a). The BAC mapping demonstrated that intrachromosomal inversions are present when comparing the X chromosome of these species, and the PNEX is similar to that found in the Capreolinae species previously studied using FISH (Frohlich et al. 2017; Proskuryakova et al. 2017; Bernegossi et al. 2022b).

The phylogenetic analyses in this study with CytB, COI, and DLoop mitochondrial genes follow the results obtained by other authors who also used CytB (Duarte et al. 2008; Heckeberg 2020; Oliveira et al. 2020) or the mitogenome (Hassanin et al. 2012; Bernegossi et al. 2022a). These clearly show the inappropriate position of *P.nemorivagus* in the genus *Mazama*. The phylogenetic position of *P.nemorivagus* in the Blastocerina subtribe also draws attention, demonstrating that this genus was one of the first to diverge from the common Blastocerina ancestor. The great distance of the genus *Passalites* from all other Odocoileina genera can also be highlighted.

Despite *S.gouazoubira* and *P.nemorivagus* having been lumped with each other several times over the course of taxonomic revisions, the species are clearly separated by different approaches (Rossi 2000; Duarte et al. 2008; Fiorillo et al. 2013; González et al. 2018), which justifies their allocation to a different genus from *Mazama* and also from each other.

Some authors suggest the possibility that *P.nemorivagus* is a cryptic species complex based on molecular (Duarte et al. 2008) and cytogenetic (Fiorillo et al. 2013) data. However, this aspect was not evaluated in this study, since we used a sample containing only specimens from the northeastern Amazon region, lacking a broader sample to solve this issue.

## ﻿Conclusions

The present study characterized a *Passalitesnemorivagus* topotype and compared it with other individuals of the same species as well as other Neotropical deer. The phylogenetic results clearly show the need to withdraw this taxon from the genus *Mazama*, since it is recovered in a phylogenetically distant clade not associated with *M.rufa*, the type species of this genus. Nevertheless, cytogenetics and morphological analyses revealed differences from the other genera of the Blastocerina subtribe, especially *Subulogouazoubira*. Thus, we validate *Passalites* as a monospecific genus with *Passalitesnemorivagus* as the type species.

## ﻿Additional information

### Author ORCIDs

Jorge Alfonso Morales-Donoso https://orcid.org/0000-0002-1684-1512

Gabrielle Queiroz Vacari https://orcid.org/0000-0003-4028-272X

Agda Maria Bernegossi https://orcid.org/0000-0003-0369-1858

Eluzai Dinai Pinto Sandoval https://orcid.org/0000-0001-6849-7373

Pedro Henrique Faria Peres https://orcid.org/0000-0002-3158-0963

David Javier Galindo https://orcid.org/0000-0003-1112-268X

Benoit de Thoisy https://orcid.org/0000-0002-8420-5112

Miluse Vozdova https://orcid.org/0000-0001-9076-8221

Svatava Kubickova https://orcid.org/0000-0002-1006-9453

José Mauricio Barbanti Duarte https://orcid.org/0000-0002-7805-0265

### Data availability

All of the data that support the findings of this study are available in the main text or Supplementary Information.

## Supplementary materials

Supplementary material 1 List of BAC clones used in *Passalitesnemorivagus* (Cuvier, 1817) topotype Data type table (docx file) Explanation note The clones were selected from the CHORI-240 cattle (BTA) library. https://binary.pensoft.net/file/866250This dataset is made available under the Open Database License (http://opendatacommons.org/licenses/odbl/1.0/). The Open Database License (ODbL) is a license agreement intended to allow users to freely share, modify, and use this Dataset while maintaining this same freedom for others, provided that the original source and author(s) are credited.Jorge Alfonso Morales-Donoso, Gabrielle Queiroz Vacari, Agda Maria Bernegossi, Eluzai Dinai Pinto Sandoval, Pedro Henrique Faria Peres, David Javier Galindo, Benoit de Thoisy, Miluse Vozdova, Svatava Kubickova, José Mauricio Barbanti Duarte

Supplementary material 2 Mitochondrial markers utilized in this study Data type table (docx file) https://binary.pensoft.net/file/866251This dataset is made available under the Open Database License (http://opendatacommons.org/licenses/odbl/1.0/). The Open Database License (ODbL) is a license agreement intended to allow users to freely share, modify, and use this Dataset while maintaining this same freedom for others, provided that the original source and author(s) are credited.Jorge Alfonso Morales-Donoso, Gabrielle Queiroz Vacari, Agda Maria Bernegossi, Eluzai Dinai Pinto Sandoval, Pedro Henrique Faria Peres, David Javier Galindo, Benoit de Thoisy, Miluse Vozdova, Svatava Kubickova, José Mauricio Barbanti Duarte

Supplementary material 3 Cranial measurements of the *Passalitesnemorivagus* specimen (T359) Data type table (docx file) Explanation note Cranial measurements of the *Passalitesnemorivagus* specimen (T359), collected in French Guiana and other male specimens of neotropical deer. Measures are in millimeters (mm) according to von den Driesch (1976). NPC = Nupecce’s museum number, TL = total length, CB L= condilobasal length, BL = basal length, SSL = short skull length, PR = premolare – prostion, BCA = basecranial axis, BFA = basefacial axis, VL = viscerocranium length, MFL = median frontal length, LN = lambda – nasal, LR= lambda – Rhinion, LP = lambda, prostion, AK = akrokranium, GLN = greatest length of nasals, SLFL = short lateral facial length, OPL = oral palatal length, LLP = lateral length of premaxilla, LCR = length of cheektooth row, LMR = length of molar row, LPR = length of premolar row, GILO = greatest inner length of orbit, GIHO = greatest inner height of orbit, GMB = greatest mastoid breadth, GBOC = greatest breadth of occipital condyles, GBBP= greatest breadth at bases of paraoccipital, GBFM = greatest breadth of foramen magnum, HFM = Height of foramen magnum: Basiom - Opisthion, GNB = Greatest neurocranium breadth, LFB = Least frontal breadth, GBAO= greatest breadth across orbits, LBBO = least breadth between orbits, ZB= zygomatic breadth, GBAN = greatest breadth across nasals, GBAP = greatest breadth across premaxillae, GPB= greatest palatal breadth, BHPSN = Basion, highest point of superior nuchal crest. N = unassigned ID number. https://binary.pensoft.net/file/866252This dataset is made available under the Open Database License (http://opendatacommons.org/licenses/odbl/1.0/). The Open Database License (ODbL) is a license agreement intended to allow users to freely share, modify, and use this Dataset while maintaining this same freedom for others, provided that the original source and author(s) are credited.Jorge Alfonso Morales-Donoso, Gabrielle Queiroz Vacari, Agda Maria Bernegossi, Eluzai Dinai Pinto Sandoval, Pedro Henrique Faria Peres, David Javier Galindo, Benoit de Thoisy, Miluse Vozdova, Svatava Kubickova, José Mauricio Barbanti Duarte

Supplementary material 4 Genetic pairwise Kimura 2 parameter distance of *Passalitesnemorivagus* compared to other Neotropical deer Data type table (docx file) https://binary.pensoft.net/file/866253This dataset is made available under the Open Database License (http://opendatacommons.org/licenses/odbl/1.0/). The Open Database License (ODbL) is a license agreement intended to allow users to freely share, modify, and use this Dataset while maintaining this same freedom for others, provided that the original source and author(s) are credited.Jorge Alfonso Morales-Donoso, Gabrielle Queiroz Vacari, Agda Maria Bernegossi, Eluzai Dinai Pinto Sandoval, Pedro Henrique Faria Peres, David Javier Galindo, Benoit de Thoisy, Miluse Vozdova, Svatava Kubickova, José Mauricio Barbanti Duarte

Supplementary material 5 Male *Passalitesnemorivagus* topotype (Cuvier, 1817) collected in French Guiana (T359) Data type figure (png file) Explanation note Ventral view of the body (A), dorsal view of the head (B), and close-up of the head view (C). https://binary.pensoft.net/file/866254This dataset is made available under the Open Database License (http://opendatacommons.org/licenses/odbl/1.0/). The Open Database License (ODbL) is a license agreement intended to allow users to freely share, modify, and use this Dataset while maintaining this same freedom for others, provided that the original source and author(s) are credited.Jorge Alfonso Morales-Donoso, Gabrielle Queiroz Vacari, Agda Maria Bernegossi, Eluzai Dinai Pinto Sandoval, Pedro Henrique Faria Peres, David Javier Galindo, Benoit de Thoisy, Miluse Vozdova, Svatava Kubickova, José Mauricio Barbanti Duarte
