# Supplementary material for: ﻿Revalidation of Passalites Gloger, 1841 for the Amazon brown brocket deer P.nemorivagus (Cuvier, 1817) (Mammalia, Artiodactyla, Cervidae)
Source: Zookeys. 2023 Jun 20;1167:241–64. doi: 10.3897/zookeys.1167.100577 (PMC10300653; doi:10.3897/zookeys.1167.100577)
Supplement: Supplementary material 1 — List of BAC clones used in Passalitesnemorivagus (Cuvier, 1817) topotype [file zookeys-1167-241_article-100577__-s001.docx]

**Supplementary Table 1**: List of BAC clones used in *Passalites nemorivagus* topotype. The clones were selected from the CHORI-240 cattle (BTA) library.

| **Chromosome** | **BAC clone** | **BTA Position (Mb)** | **BAC clone** | **BTA Position (Mb)** | **BAC clone** | **BTA Position (MB)** | **BAC clone** | **BTA Position (Mb)** | **BAC clone** | **BTA Position (Mb)** | | | |
| --- | --- | --- | --- | --- | --- | --- | --- | --- | --- | --- | --- | --- | --- |
| BTA1 | 106N15 | 2.08-2.25 | 69G2 | 57.29-57,48 | 109I18 | 116,68-116.91 |  |  | 273F5 | 154.36-154.55 | | | |
| BTA2 | 42D15 | 9.51-9.71 | 110M8 | 44.41-44,65 | 124N14 | 96.51-96.71 |  |  | 437C7 | 135.51- 135.71 | | | |
| BTA3 | 24H18 | 4.38-4.62 | 274A19 | 53.24-53.51 | 106P15 | 98.75-98.97 |  |  | 433N8 | 121.25-121.43 | | | |
| BTA4 | 168K3 | 5.43-5.62 | 215P21 | 40.28-40.51 | 95A8 | 77.04-77.23 |  |  | 259C9 | 119.29-119.48 | | | |
| BTA5 | 411D6 | 5.81-5.99 | 56D20 | 55.50-55.75 | 59F16 | 68.51-68,70 | 78L8 | 70.86-71.06 | 100C21 | 119.70-119.89 | | | |
| BTA6 | 89B23 | 3.37-3.55 | 46O3 | 61.12-61.34 | 45E5 | 64.74-64.97 | 66P17 | 70.43-70.66 | 200F18 | 117.34-117.56 | | | |
| BTA7 | 57O13 | 1.36-1.56 | 117P3 | 38.88-39.13 | 57A5 | 81.18 -81.42 |  |  | 105G13 | 109.52-109.76 | | | |
| BTA8 | 418M12 | 1.50-1.67 | 71G4 | 62.73-62.93 | 223P21 | 63.95-64.13 | 512A24 | 69.30-69.50 | 504A4 | 112.10-112.29 | | | |
| BTA9 | 448F18 | 9.19-9.39 | 78C10 | 60.39-60.60 | 64B22 | 63.97-64.19 |  |  | 90A6 | 103.38-103.59 | | | |
| BTA10 | 173B8 | 3.97-4.21 | 131N22 | 42.65-42.86 | 90G7 | 76.15-76.37 |  |  | 214M18 | 103.02-103.24 | | | |
| BTA11 | 98M8 | 3.70-3.92 | 91N11 | 37.25-37.46 | 164D13 | 72.21-72.43 |  |  | 463C19 | 106.02-106.22 | | | |
| BTA12 | 68F22 | 11.10-11.33 | 27O21 | 36.43-36.65 | 115C4 | 86.36-86.58 |  | |  |  | |  | |
| BTA13 | 174M6 | 7.23-7.44 | 114C2 | 42.80-43.05 | 278J11 | 75.01-75.26 |  |  |  |  | |  | |
| BTA15 | 122B6 | 5.29-5.51 | 121K12 | 41.87-42.09 | 399A11 | 77.08-77.28 |  |  |  |  | |  | |
| BTA16 | 283F4 | 2.65-2.86 | 124O11 | 38.84-39.04 | 140I17 | 80.23-80.49 |  |  |  |  | |  | |
| BTA17 | 63H8 | 3.90-4.12 | 215C16 | 35.26-35.46 | 98F22 | 72.19-72.41 |  |  |  |  | |  | |
| BTA18 | 121P7 | 2.76-2.96 | 79A23 | 34.69-34.92 | 105L6 | 65.58-65.76 |  |  |  |  | |  | |
| BTA19 | 130E10 | 9.12-9.34 | 50L8 | 34.35-34.57 | 188A20 | 55.91-56.09 |  |  |  |  | |  | |
| BTA20 | 189H8 | 5.16-6.89 | 60G20 | 35.32-35.51 | 378K8 | 71.13-71.32 |  |  |  |  | |  | |
| BTA21 | 95M7 | 5.45-5.66 | 377F11 | 33.80-33.98 | 283I6 | 66.79-67.00 |  |  | **Chromosome** | | **BAC clone** | | **BTA position (Mb)** |
| BTA22 | 153G8 | 1.61-1.82 | 168H4 | 38.93-39.12 | 155B17 | 59.06-59.29 |  |  | BTAXp | | 159O16 | | 23.04-23.22 |
| BTA23 | 194A20 | 7.27-7.48 | 77G24 | 28.26-28.47 | 166B3 | 50.80-51.03 |  |  |  |  | 67P21 | | 33.77-34.00 |
| BTA24 | 205G22 | 0.61-0.82 | 90M14 | 32.92-33.16 | 453P2 | 61.93-62.12 |  |  | BTAXqprox | | 311B9 | | 47.76-47.97 |
| BTA25 | 93C17 | 3.86-4.09 | 89A17 | 21.27-21.51 | 124M6 | 37.22-37.43 |  |  |  |  | 93K24 | | 57.73-57.95 |
| BTA26 | 205E12 | 8.51-8.68 | 47G11 | 24.54-24.77 | 457G23 | 49.25-49.41 |  |  |  |  | 316D2 | | 68.49-68.68 |
| BTA27 | 79A12 | 1.27-1.52 | 41E12 | 26.81-27.00 | 126M6 | 41.90-42.14 |  |  | BTAXqdist | | 40H2 | | 74.95-75.12 |
| BTA28 | 321F10 | 1.40-1.59 | 108O21 | 24.74-24.94 | 361P13 | 44.02-44.18 |  |  | BTAXPAR | | 453C5 | | 144.43-144.62 |
| BTA29 | 384F12 | 5.79-5.96 | 103L15 | 28.99-29.16 | 472F18 | 39.69-39.85 |  |  |  |  | 326C13 | | 148.27-148.47 |
