# Supplementary material for: ﻿Revalidation of Passalites Gloger, 1841 for the Amazon brown brocket deer P.nemorivagus (Cuvier, 1817) (Mammalia, Artiodactyla, Cervidae)
Source: Zookeys. 2023 Jun 20;1167:241–64. doi: 10.3897/zookeys.1167.100577 (PMC10300653; doi:10.3897/zookeys.1167.100577)
Supplement: Supplementary material 2 — Mitochondrial markers utilized in this study [file zookeys-1167-241_article-100577__-s002.docx]

**Table S2.** Mitochondrial markers used in this study.

| **Genes** | **size (pb)** | **Anealing Tº** | **Reference** | **Primers** | **Sequence (5´3´)** |
| --- | --- | --- | --- | --- | --- |
| Cyt B | 480  660 | 54ºC | Kocher *et al*., 1989  Duarte *et al*., 2008 | L14124  H15149  FARH  FARL | (F) AAAAAGCTTCCATCCAACATCTCAGCATGATGAAA  (R)AAACTGCAGCCCCTCAGAATGATATTTGTCCTCA.  (F)TCCAATAGTAATAAAGGGGTGTTCA  (R)CCATGAGGACAAATATCATTCTGTA |
| COI | 658 | 54º C | Folmer *et al*., 1994 | LCO1490  HCO2198 | (F)GGTCAACAAATCATAAAGATATTGG  (R)TAAACTTCAGGGTGACCAAAAAATA |
| D- loop | 690 | 52ºC | Vilá *et al*., 1999 | DL  THR | (F)CAATTCCCGGTCTTGTAAACC  (R)CCTGAAGTAGGAACCAGATG |
