# Supplementary material for: ﻿Revalidation of Passalites Gloger, 1841 for the Amazon brown brocket deer P.nemorivagus (Cuvier, 1817) (Mammalia, Artiodactyla, Cervidae)
Source: Zookeys. 2023 Jun 20;1167:241–64. doi: 10.3897/zookeys.1167.100577 (PMC10300653; doi:10.3897/zookeys.1167.100577)
Supplement: Supplementary material 3 — Cranial measurements of the Passalitesnemorivagus specimen (T359) [file zookeys-1167-241_article-100577__-s003.docx]

**Table S3:** Cranial measurements of the *Passalites nemorivagus* T359, collected in French Guiana and other neotropical deer males, represented in millimeters (mm) according to DRIESCH (1976).

| Species | NPC | ID | CT | CCB | CB | CCC | PP | EBC | EBF | CVC | CFM | LN | LR | LP | ACR | MCN | CCFL | CPO | CLP | DCD | CLM |
| --- | --- | --- | --- | --- | --- | --- | --- | --- | --- | --- | --- | --- | --- | --- | --- | --- | --- | --- | --- | --- | --- |
| *P. nemorivagus (Topotype)* | 80 | T359 | 172.41 | 165.86 | 148.85 | 99.59 | 48.84 | 26.41 | 120.65 | 77.87 | 105.33 | 92.01 | 140.00 | 172.45 | 111.22 | 50.16 | 80.44 | 75.43 | 38.35 | 50.78 | 28.77 |
| *P. nemorivagus* | 88 | T295 | 175.63 | 171.01 | 155.66 | 103.42 | 49.80 | 22.38 | 130.08 | 83.94 | 97.04 | 86.28 | 136.21 | 165.99 | 127.57 | 48.71 | 85.28 | 75.63 | 37.31 | 56.90 | 32.96 |
| *P. nemorivagus* | 165 | CATFAP4 | 178.55 | 173.37 | 155.97 | 101.31 | 55.39 | 21.31 | 129.50 | 83.22 | 104.70 | 91.81 | 139.98 | 168.21 | 126.89 | 50.60 | 89.43 | 76.15 | 44.61 | 52.09 | 29.27 |
| *P. nemorivagus* | 108 | T 371 | 171.07 | 165.22 | 149.92 | 100.62 | 50.17 | 23.70 | 124.99 | 79.59 | 102.34 | 89.09 | 137.71 | 171.55 | 120.79 | 51.78 | 78.28 | 78.40 | 42.30 | 48.29 | 27.54 |
| *P. nemorivagus* | 6 | T264 | 162.87 | 158.10 | 142.34 | 103.63 | 48.30 | 27.61 | 129.20 | 85.53 | 101.8 | 107.81 | 137.40 | 172.38 | 126.55 | 45.36 | 80.30 | 76.81 | 46.81 | 55.15 | 35.71 |
| *P. nemorivagus* | 53 | T265 | 182.92 | 172.08 | 160.40 | 102.19 | 57.25 | 29.41 | 130.34 | 81.46 | 101.93 | 103.74 | 142.5 | 172.93 | 125.84 | 51.38 | 84.01 | 76.18 | 41.88 | 57.18 | 43.21 |
| *S. gouazoubira* | 57 | T347B | 177.90 | 170.04 | 157.18 | 104.88 | 55.01 | 24.82 | 134.67 | 78.59 | 108.10 | 96.52 | 145.37 | 168.93 | 126.34 | 51.38 | 88.12 | 71.12 | 41.05 | 51.03 | 27.57 |
| *S. gouazoubira* | 154 | T 403 | 180.68 | 172.86 | 154.24 | 99.92 | 55.21 | 24.71 | 130.25 | 77.07 | 116.27 | 106.67 | 147.80 | 174.95 | 129.59 | 47.88 | 90.05 | 76.05 | 40.91 | 51.61 | 27.90 |
| *S. gouazoubira* | 61 | T323 | 176.02 | 162.93 | 142.31 | 103.33 | 54.02 | 22.31 | 122.65 | 75.03 | 102.34 | 99.93 | 141.13 | 175.51 | 123.24 | 41.57 | 89.07 | 73.65 | 39.91 | 40.19 | 26.57 |
| *S. gouazoubira* | 139 | T409 | 170.82 | 163.95 | 140.39 | 94.55 | 56.78 | 26.47 | 123.43 | 75.04 | 105.26 | 98.6 | 141.03 | 164.57 | 122.32 | 49.91 | 72.04 | 80.21 | 47.12 | 55.77 | 30.75 |
| *S. gouazoubira (Neotype)* | 113 | T377 | 165.91 | 157.14 | 147.02 | 102.44 | 46.25 | 19.67 | 127.19 | 75.68 | 99.87 | 91.09 | 128.70 | 156.21 | 117.51 | 44.20 | 87.65 | 66.62 | 41.30 | 43.13 | 26.86 |
| *M. americana (Carajás)* | 110 | T274 | 220.74 | 219.45 | 187.43 | 121.52 | 68.13 | 27.26 | 172.07 | 104.72 | 127.52 | 103.53 | 175.89 | 212.73 | 152.61 | 65.07 | 116.10 | 102.61 | 61.17 | 63.42 | 34.39 |
| *M. americana (Neotype)* | 79 | T358 | 223.19 | 222.99 | 197.33 | 137.02 | 61.87 | 27.27 | 170.50 | 119.03 | 115.38 | 103.44 | 172.54 | 212.02 | 151.73 | 71.00 | 116.10 | 99.14 | 61.17 | 68.87 | 37.77 |
| *M. americana (Santarém)* | 18 | T260 | 205.90 | 210.68 | 187.21 | 122.07 | 63.91 | 32.89 | 151.11 | 104.22 | 120.79 | 107.58 | 170.22 | 206.04 | 139.55 | 60.63 | 101.07 | 98.18 | 58.23 | 54.29 | 32.8 |
| *M. americana (Santarém)* | 78 | T259 | 225.41 | 216.91 | 191.54 | 124.01 | 68.85 | 33.57 | 165.91 | 106.8 | 129.17 | 117.77 | 188.12 | 214.19 | 155.62 | 64.31 | 120.90 | 102.88 | 60.90 | 60.76 | 31.35 |
| *M. americana (Juína)* | 147 | T247 | 220.18 | 209.83 | 192.99 | 124.24 | 66.41 | 33.57 | 159.57 | 103.05 | 124.42 | 107.49 | 172.49 | 205.13 | 152.64 | 67.49 | 113.14 | 96.43 | 51.82 | 60.61 | 33.30 |
| *M. americana (Juína)* | 7 | T251 | 210.53 | 209.02 | 189.66 | 125.24 | 66.41 | 37.52 | 153.12 | 104.62 | 117.97 | 106.62 | 172.27 | 204.81 | 144.87 | 66.18 | 111.23 | 87.92 | 59.10 | 65.40 | 38.23 |
| *M. americana (Rondônia)* | 19 | T269 | 210.62 | 206.04 | 190.18 | 129.89 | 64.54 | 33.63 | 157.99 | 94.91 | 125.30 | 116.93 | 166.54 | 201.84 | 143.76 | 53.35 | 107.15 | 93.01 | 48.22 | 64.11 | 35.76 |
| *M. americana (Rondônia)* | 55 | T206 | 215.31 | 204.88 | 191.83 | 123.30 | 67.03 | 31.01 | 162.16 | 100.89 | 125.13 | 114.63 | 177.74 | 207.81 | 146.70 | 64.26 | 110.15 | 95.39 | 54.28 | 63.47 | 37.41 |
| *M. rufa* | 126 | T205 | 221.61 | 209.37 | 194.46 | 122.72 | 62.39 | 43.28 | 153.49 | 97.64 | 128.45 | 125.35 | 183.18 | 210.53 | 139.78 | 59.19 | 116.88 | 101.87 | 51.33 | 63.2 | 32.17 |
| *M. rufa* | 34 | T268 | 216.69 | 214.96 | 190.88 | 114.68 | 72.11 | 29.40 | 157.87 | 103.39 | 120.91 | 106.31 | 171.44 | 204.73 | 148.53 | 64.38 | 109.92 | 101.53 | 49.55 | 60.78 | 35.04 |
| *M. jucunda* | 141 | T412 | 196.01 | 195.75 | 178.24 | 112.45 | 66.69 | 29.40 | 143.23 | 96.50 | 120.50 | 106.50 | 164.78 | 194.23 | 140.64 | 56.69 | 102.03 | 95.08 | 56.57 | 60.54 | 34.11 |
| *M. jucunda* | 1 | T340 | 205.1 | 191.54 | 176.47 | 118.67 | 62.11 | 32.01 | 149.45 | 88.34 | 119.5 | 116.4 | 167.89 | 192.54 | 138.82 | 55.09 | 101.54 | 81.03 | 49.56 | 55.92 | 30.87 |
| *M. nana* | 144 | T304 | 166.7 | 160.40 | 147.55 | 97.68 | 47.91 | 30.81 | 112.31 | 73.62 | 104.23 | 90.42 | 137.37 | 157.33 | 119.67 | 47.16 | 77.45 | 68.84 | 38.76 | 48.00 | 26.34 |
| *M. nana* | 114 | N | 163.7 | 149.9 | 130.01 | 95.3 | 51.02 | 21.4 | 124.45 | 72.09 | 103.89 | 98.88 | 144.52 | 161.17 | 117.06 | 48.45 | 80.02 | 72.51 | 34.65 | 49.02 | 26.80 |

| Species | NPC | ID | CLP2 | MCIO | MAIO | MLM | MLPP | MLFM | AFM | MLNC | LFM | MLEO | LMEO | LZ | MLEN | MLPM | MLP | BCNS |
| --- | --- | --- | --- | --- | --- | --- | --- | --- | --- | --- | --- | --- | --- | --- | --- | --- | --- | --- |
| *P. nemorivagus (Topotype)* | 80 | T359 | 24.21 | 31.59 | 32.54 | 57.56 | 45.97 | 13.50 | 17.93 | 52.31 | 43.25 | 66.24 | 44.55 | 71.67 | 15.97 | 16.45 | 49.88 | 41.21 |
| *P. nemorivagus* | 88 | T295 | 26.65 | 32.97 | 33.62 | 56.55 | 51.47 | 16.65 | 17.52 | 58.37 | 58.06 | 72.27 | 52.09 | 76.57 | 21.60 | 23.48 | 81.71 | 43.57 |
| *P. nemorivagus* | 165 | CATFAP4 | 24.68 | 32.56 | 32.20 | 57.56 | 47.93 | 15.16 | 18.40 | 57.94 | 57.06 | 63.87 | 49.78 | 76.45 | 19.85 | 21.75 | 76.44 | 40.70 |
| *P. nemorivagus* | 108 | T371 | 20.81 | 30.50 | 31.63 | 64.73 | 43.10 | 15.34 | 14.80 | 52.31 | 42.61 | 64.37 | 45.16 | 68.03 | 20.83 | 17.82 | 49.62 | 37.52 |
| *P. nemorivagus* | 6 | T264 | 23.39 | 32.75 | 35.68 | 55.5 | 46.79 | 13.50 | 17.93 | 54.36 | 43.18 | 60.05 | 45.04 | 72.91 | 15.97 | 18.33 | 46.98 | 42.62 |
| *P. nemorivagus* | 53 | T265 | 24.21 | 35.77 | 32.54 | 64.73 | 48.78 | 15.34 | 15.11 | 51.94 | 48.66 | 68.08 | 49.10 | 71.71 | 20.16 | 20.50 | 52.79 | 40.94 |
| *S. gouazoubira* | 57 | T347B | 22.53 | 26.78 | 28.59 | 52.60 | 46.89 | 15.03 | 14.22 | 51.67 | 50.83 | 62.73 | 43.72 | 74.00 | 17.39 | 20.50 | 51.81 | 46.99 |
| *S. gouazoubira* | 154 | T403 | 22.46 | 28.91 | 28.90 | 58.91 | 53.22 | 15.62 | 17.07 | 56.74 | 62.79 | 70.19 | 48.22 | 79.58 | 21.96 | 23.60 | 50.93 | 46.32 |
| *S. gouazoubira* | 61 | T323 | 22.9 | 29.51 | 30.51 | 58.69 | 47.42 | 19.14 | 16.62 | 52.14 | 43.80 | 70.09 | 46.52 | 78.09 | 18.02 | 22.03 | 53.02 | 43.35 |
| *S. gouazoubira* | 139 | T409 | 24.32 | 30.33 | 29.07 | 54.65 | 51.92 | 15.58 | 15.07 | 53.04 | 43.65 | 67.35 | 41.23 | 77.65 | 22.03 | 25.41 | 53.09 | 46.13 |
| *S. gouazoubira (Neotype)* | 113 | T377 | 27.02 | 28.46 | 26.55 | 55.98 | 37.99 | 14.37 | 15.05 | 51.04 | 43.87 | 54.29 | 40.49 | 73.67 | 15.90 | 20.83 | 52.18 | 38.31 |
| *M. americana (Carajás)* | 110 | T274 | 30.86 | 31.44 | 39.67 | 69.32 | 57.62 | 18.73 | 15.83 | 59.80 | 68.34 | 80.93 | 57.12 | 97.93 | 23.06 | 27.67 | 61.27 | 51.06 |
| *M. americana (Neotype)* | 79 | T358 | 30.16 | 30.22 | 37.31 | 66.28 | 60.14 | 18.18 | 19.18 | 63.13 | 66.65 | 84.59 | 46.16 | 100.97 | 21.35 | 28.10 | 70.44 | 50.42 |
| *M. americana (Santarém)* | 18 | T260 | 26.77 | 32.75 | 36.07 | 67.34 | 59.29 | 15.96 | 18.22 | 62.17 | 63.9 | 81.53 | 51.89 | 87.46 | 20.36 | 24.05 | 60.89 | 49.77 |
| *M. americana (Santarém)* | 78 | T259 | 29.22 | 35.33 | 39.19 | 67.09 | 58.28 | 15.28 | 18.05 | 64.75 | 68.92 | 82.16 | 51.71 | 92.31 | 23.76 | 24.88 | 66.72 | 69.38 |
| *M. americana (Juína)* | 147 | T247 | 30.42 | 33.29 | 37.32 | 64.82 | 62.11 | 19.18 | 18.29 | 65.53 | 66.34 | 79.44 | 55.75 | 89.36 | 26.13 | 31.24 | 66.72 | 50.4 |
| *M. americana (Juína)* | 7 | T251 | 30.34 | 35.36 | 36.12 | 64.33 | 62.12 | 19.53 | 19.17 | 62.10 | 68.98 | 82.65 | 54.92 | 91.78 | 17.44 | 22.61 | 60.79 | 61.28 |
| *M. americana (Rondônia)* | 19 | T269 | 29.22 | 32.81 | 33.98 | 67.72 | 59.10 | 15.79 | 15.02 | 61.65 | 72.33 | 83.04 | 53.87 | 92.42 | 21.94 | 26.04 | 64.80 | 73.30 |
| *M. americana (Rondônia)* | 55 | T206 | 28.15 | 35.08 | 41.18 | 60.37 | 55.98 | 18.41 | 19.89 | 63.01 | 69.69 | 80.05 | 56.15 | 92.07 | 23.35 | 24.92 | 53.74 | 69.68 |
| *M. rufa* | 126 | T205 | 28.73 | 33.58 | 37.55 | 60.91 | 55.6 | 20.54 | 20.03 | 58.98 | 66.3 | 81.82 | 54.22 | 98.99 | 22.97 | 27.44 | 61.06 | 67.51 |
| *M. rufa* | 34 | T268 | 30.78 | 36.65 | 38.45 | 70.24 | 56.89 | 14.08 | 21.97 | 65.87 | 74.22 | 82.68 | 52.98 | 100.48 | 28.88 | 37.22 | 67.39 | 49.84 |
| *M. jucunda* | 141 | T412 | 29.60 | 33.32 | 32.82 | 62.54 | 55.08 | 17.44 | 17.14 | 59.96 | 64.16 | 77.03 | 51.84 | 89.42 | 23.02 | 32.41 | 62.80 | 57.59 |
| *M. jucunda* | 1 | T340 | 24.6 | 31.07 | 31.65 | 68.02 | 53.01 | 19.5 | 17.54 | 57.08 | 56.71 | 76.66 | 50.12 | 81.92 | 17.32 | 24.01 | 56.02 | 47.61 |
| *M. nana* | 144 | T304 | 22.44 | 30.39 | 34.16 | 54.20 | 49.31 | 17.04 | 15.32 | 57.20 | 54.64 | 65.01 | 44.04 | 75.89 | 21.42 | 23.25 | 50.15 | 44.03 |
| *M. nana* | 114 | N | 22.95 | 28.53 | 27.77 | 52.54 | 47.46 | 21.23 | 16.4 | 50.92 | 48.09 | 66.23 | 43.62 | 71.98 | 16.18 | 19.42 | 51.76 | 40.37 |

NPC= Nupecce’s museum number, TL= total length, CBL= condilobasal length, BL= basal length, SSL= short skull length, PR= premolare – prostion, BCA= basecranial axis, BFA= basefacial axis, VL= viscerocranium length, MFL= median frontal length, LN= lambda – nasal, LR= lambda – Rhinion, LP= lambda – prostion , AK= akrokranium, GLN= greatest length of the nasals, SLFL= short lateral facial length, OPL= oral palatal length, LLP= lateral length of the premaxilla, LCR= length of the cheektooth row, LMR= length of the molar row, LPR= length of the premolar row, GILO= greatest inner length of the orbit, GIHO= greatest inner height of the orbit, GMB= greatest mastoid breadth, GBOC= greatest breadth of the occipital condyles, GBBP= greatest breadth at the bases of the paraoccipital, GBFM= greatest breadth of the foramen magnum, HFM= Height of the foramen magnum: Basiom - Opisthion, GNB= Greatest neurocranium breadth, LFB= Least frontal breadth, GBAO= greatest breadth across the orbits, LBBO= least breadth between the orbits, ZB= zygomatic breadth, GBAN= greatest breadth across the nasals, GBAP= greatest breadth across the premaxillae, GPB= greatest palatal breadth, BHPSN= Basion – The highest point of the superior nuchal crest. N= unassigned ID number.
