# Supplementary material for: ﻿Revalidation of Passalites Gloger, 1841 for the Amazon brown brocket deer P.nemorivagus (Cuvier, 1817) (Mammalia, Artiodactyla, Cervidae)
Source: Zookeys. 2023 Jun 20;1167:241–64. doi: 10.3897/zookeys.1167.100577 (PMC10300653; doi:10.3897/zookeys.1167.100577)
Supplement: Supplementary material 4 — Genetic pairwise Kimura 2 parameter distance of Passalitesnemorivagus compared to other Neotropical deer [file zookeys-1167-241_article-100577__-s004.docx]

**Table S4.** Genetic pairwise Kimura 2 parameter distance of *Passalites nemorivagus* compared to other Neotropical deer.

|  | *Ozotoceros bezoarticus* | *Blastoceros dichotomus* | *Passalites nemorivagus* | *Subulo gouazoubira* | *Mazama nana* | *Mazama jucunda* | *Mazama americana* | *Mazama temama* |
| --- | --- | --- | --- | --- | --- | --- | --- | --- |
| *Blastoceros dichotomus* | 0,0966 |  |  |  |  |  |  |  |
| *Passalites nemorivagus* | 0,1246 | 0,1278 |  |  |  |  |  |  |
| *Subulo gouazoubira* | 0,0998 | 0,0885 | 0,1223 |  |  |  |  |  |
| *Mazama nana* | 0,1280 | 0,1260 | 0,1344 | 0,1259 |  |  |  |  |
| *Mazama jucunda* | 0,1266 | 0,1314 | 0,1362 | 0,1291 | 0,0222 |  |  |  |
| *Mazama americana* | 0,1308 | 0,1330 | 0,1395 | 0,1287 | 0,0441 | 0,0465 |  |  |
| *Mazama temama* | 0,1277 | 0,1329 | 0,1477 | 0,1299 | 0,0648 | 0,0673 | 0,0671 |  |
| *outgroup* | 0,1514 | 0,1415 | 0,1571 | 0,1464 | 0,1384 | 0,1422 | 0,1435 | 0,1442 |
