# Supplementary figures and images for: ﻿Revalidation of Passalites Gloger, 1841 for the Amazon brown brocket deer P.nemorivagus (Cuvier, 1817) (Mammalia, Artiodactyla, Cervidae)
Source: Zookeys. 2023 Jun 20;1167:241–64. doi: 10.3897/zookeys.1167.100577 (PMC10300653; doi:10.3897/zookeys.1167.100577)

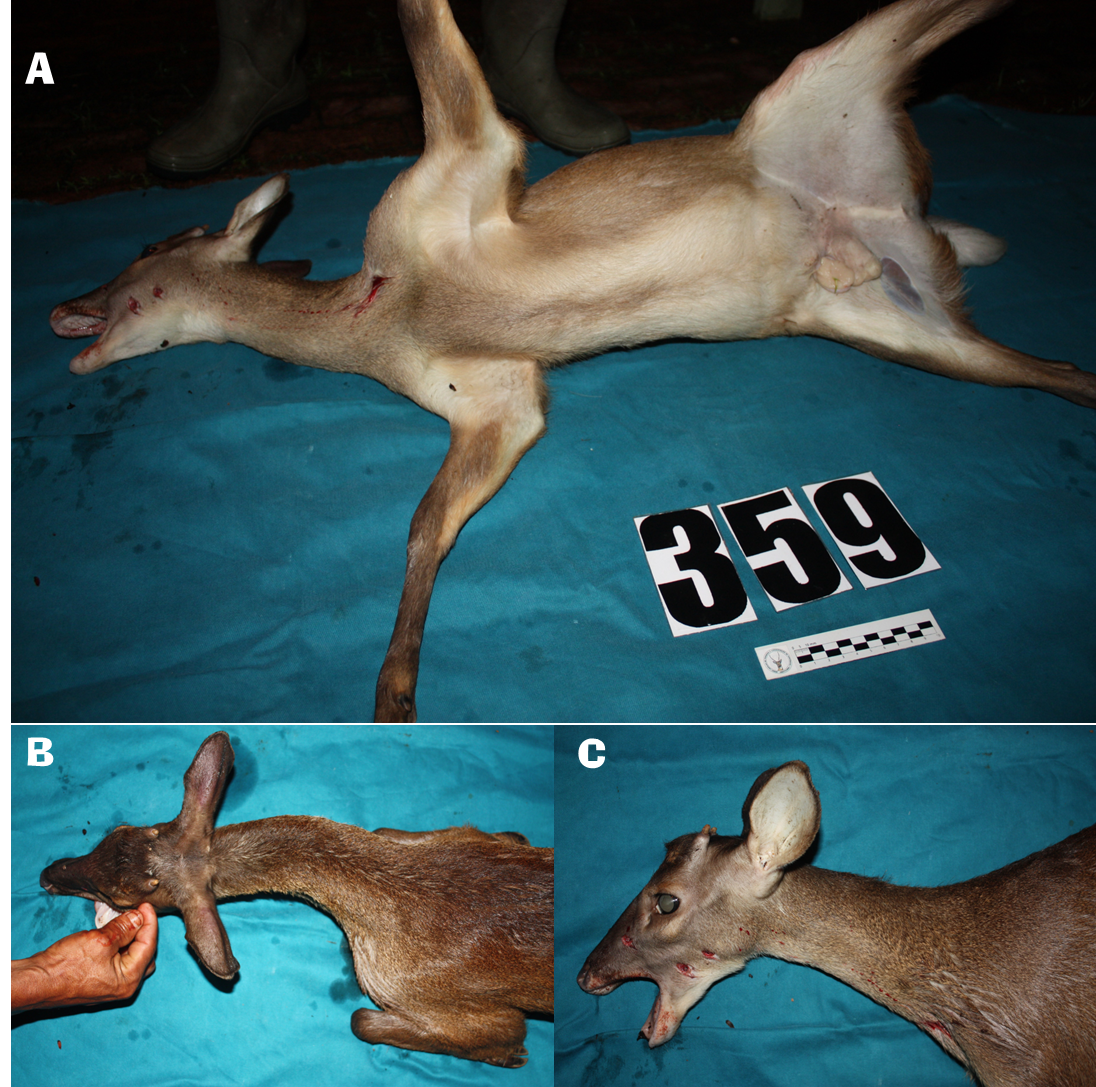

Supplement: Supplementary material 5 — Male Passalitesnemorivagus topotype (Cuvier, 1817) collected in French Guiana (T359) [file zookeys-1167-241_article-100577__-s005.png]
